# Serotonergic system in hypoxic ventilatory response in unilateral rat model of Parkinson’s disease

**DOI:** 10.1186/s12929-017-0331-2

**Published:** 2017-03-27

**Authors:** Kryspin Andrzejewski, Katarzyna Kaczyńska, Małgorzata Zaremba

**Affiliations:** 1 Laboratory of Respiration Physiology, Mossakowski Medical Research Centre Polish Academy of Sciences, Pawińskiego 5, 02-106 Warsaw, Poland; 2 Department of Experimental and Clinical Pharmacology, Centre for Preclinical Research (CePT), Medical University of Warsaw, Warsaw, Poland

**Keywords:** 6-OHDA rat model, Hypoxia, Serotonergic 5-HT_2_ receptors, DOI, Ketanserin

## Abstract

**Background:**

Malfunctioning of the serotonergic system in Parkinson’s disease may contribute to non-motor symptoms such as respiratory complications. Thus the aim of our study was to investigate the role of serotonin 5-HT_2_ receptors in the modulation of normoxic breathing and the hypoxic ventilatory response (HVR) in rat model of Parkinson’s disease.

**Methods:**

Wistar rats were lesioned unilaterally with double 6-hydroxydopamine (6-OHDA) injection to the right medial forebrain bundle (MFB). Before lesion and two weeks later animals were put in whole body plethysmography chamber and exposed to hypoxia (8% O_2_). Before hypoxic tests animals received intraperitoneal injections of DOI and ketanserin. Efficacy of lesion was confirmed by cylinder test, assessing limb use asymmetry.

**Results:**

Degeneration of the nigrostriatal pathway augmented response of tidal volume and minute ventilation to hypoxia. DOI administration in control and lesion state caused a significant rise in normoxic respiratory rate and minute ventilation. Yet, ventilatory response of these parameters to hypoxia was attenuated. Post-DOI magnitude of HVR in lesioned state was decreased in compare to pre-lesion control. Subsequent ketanserin injection reverted DOI-induced respiratory effects. We demonstrated that 6-OHDA treatment decreased the content of serotonin in the injured striatum and on both sides of the brainstem, leaving the concentration of noradrenaline on unchanged level.

**Conclusions:**

These observations showed that damage of the nigrostriatal system initiates changes in the serotonergic system, confirmed by reduced concentration of serotonin in the striatum and brainstem, which affects the magnitude of respiratory response to hypoxia after activation of 5-HT_2_ receptors.

## Background

Parkinson’s disease (PD), one of the most frequent neurodegenerative diseases appearing in an old age, is characterized by the progressive loss of dopamine (DA) neurons in substantia nigra pars compacta (SN), leading to striatal DA depletion [1, 2]. Apart from the motor deficits patients with PD frequently exhibit non-motor symptoms, which although significant are still often undiagnosed and untreated [3]. Among the latter are respiratory disturbances: restrictive pulmonary function, respiratory dysrhythmias and upper airway dysfunction [4, 5]. Ventilatory response to hypoxia (HVR) has been examined in very few human studies, which showed quite divergent results: enhanced response [6], diminished [7, 8] and unchanged [9]. Latest animal research on experimental PD models displayed mainly increased response of tidal volume and attenuated response of frequency of breathing to hypoxic stimulus, ascribed mainly to dopamine brain depletion evoked by 6-hydroxydopamine [10] and reserpine administration [11]. However, besides changes in dopaminergic neurotransmission serotonergic system undergoes degeneration in PD. Dystrophy and loss of serotonin producing neurons has been observed in medullary raphe [12] connected with brainstem regions of respiratory network control like solitary tract nucleus (NTS) and Botzinger and pre-Botzinger complex [13, 14].

Serotonin (5-HT) is a monoamine neurotransmitter, engaged in stimulatory neuromodulation of the respiratory rhythm [15, 16]. Abnormal level of 5-HT metabolites in the cerebrospinal fluid is considered to be linked with attenuated ventilatory response to hypoxia observed in Prader-Willi syndrome [14].

It has been confirmed that serotonin modulates dynamics of hypoxic ventilatory response [17]. Moreover, stimulatory 5HT_2_ receptors in dorsomedial medulla oblongata have been considered to be responsible for initiation of hypoxic hyperventilation [18]. Chemical lesion of the raphe magnus, where serotonergic neurons are present, produced increased ventilatory response to hypoxia in awake rats [19]. Similarly, extensive damage of medullary 5-HT neurons in newborn piglets augmented respiratory rate response to hypoxia during sleep [20].

We presume, that altered 5-HT neurotransmission in PD [12] may be one of the reasons of respiratory dysfunctions present under normoxic and hypoxic conditions. Therefore our first goal was the examination of serotonin concentration in the striatum and brainstem of the rats injected with 6-hydroxydopamine (6-OHDA) into the medial forebrain bundle (MFB). Next, we investigated the role of serotonin and serotoninergic 5HT_2_ receptors in hypoxic ventilatory response in 6-OHDA treated rats. This was achieved by intraperitoneal administration before each hypoxic test DOI and ketanserin, 5HT_2_ receptor agonist and antagonist, respectively.

## Methods

### Animals and experimental protocol

All experimental procedures were approved by the IV Local Ethical Committee for Animal Experimentation (Warsaw, Poland) and conducted in accordance with the international/EU guidelines and regulations on the use and care of laboratory animals. Young adult male Wistar rats from MMRC PAS (Warsaw, Poland) weighing 230–260 g at the beginning of the experiment were housed under standard laboratory conditions with a 12 h light/12 h dark cycle and unrestricted access to food and water. Unilateral injection to the right MFB with 6-hydroxydopamine (*n* = 9) or vehicle (*n* = 9) was performed. All rats were investigated for hypoxic ventilatory response before and fourteen days after injection. Sham operation was aimed to show that surgical operation itself and injection of the vehicle does not produce striatal depletion of monoamines, motor deficits and changes in normoxic and hypoxic breathing. Animals lesioned with 6-OHDA after first control hypoxia were treated intraperitoneally (i.p.) with DOI (2, 5-dimethoxy-4-iodoamphetamine) at a dose of 1.5 mg kg^−1^ and after 30 min they were exposed to second hypoxic test. Then animals received i.p. administration of ketanserin at a dose of 1 mg kg^−1^ and 30 min later last hypoxic test was taken up. Each rat served as its own control, thus the agonist and antagonist were injected intraperitoneally twice in each rat; before and after 6-OHDA lesion. Period of 30 s breathing preceding hypoxia was considered as a control normoxic breathing. Three min period of hypoxic breathing was divided on 30 s time periods and analyzed.

**Fig. 1:**
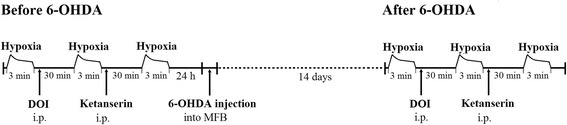
Scheme of the experimental protocol in 6-OHDA lesioned rats

Experimental groups and paradigm:

I. sham operation and vehicle injection to the MFB; hypoxic test before and two weeks after surgery
II. lesion with 6-OHDA to the MFB; control hypoxic test + hypoxic test after DOI + hypoxic test after ketanserin, prior to and two weeks after injury (Fig. 1).

### Surgery and 6-OHDA lesion

Rats were anesthetized with an intraperitoneal injection of thiopentalum natricum (Sandoz GmbH, Austria) at a dose of 90 mg kg^−1^. To prevent the uptake of 6-OHDA by noradrenergic nerve terminals, 30 min before the operation rats received i.p. administration of desipramine hydrochloride (25 mg kg^−1^, Sigma Aldrich, Poland). After positioning of the animal in a stereotaxic instrument (Digital Lab Standard Stereotaxic Stoelting, USA) the skin was incised, the skull was trephined with a dental drill in a specific stereotaxic coordinates. Vehicle or 6-hydroxydopamine hydrochloride (4 μg μl^−1^ dissolved in 0.9% NaCl containing 0.1% ascorbic acid (Sigma Aldrich, Poland)) was injected with a sterile Hamilton micro syringe at a volume of 5 μl (rate of 1 μl min^−1^) in two locations of the MFB. After injection, the needle was left in the brain for 5 min to prevent the solution from flowing backward and then was slowly retracted. Stereotaxic coordinates for the first injection site were: antero-posterior, bregma: −2.2 mm, lateral: +1.5 mm, ventral dura: −7.8 mm, and incisor bar: −3.5 mm, and second site: antero-posterior, bregma: −4.4 mm, lateral: +1.5 mm, ventral dura: −7.9 mm, and incisor bar: −3.5 mm. After the surgery, rats were left to recover with standard laboratory conditions, and unlimited access to food and water.

### Ventilatory recordings

Ventilatory measurements were performed in a whole body rodent plethysmograph (WBP, model PLY 3223, Buxco Electronics, USA). The system is composed of two chambers: recording and reference. The pressure fluctuations, created by the inspiration and expiration of the animal in the experimental chamber, are proportional to respiratory flow. Pressure changes within the chamber are the result of gas compression caused by pressure changes in the thoracic gas producing inspiratory and expiratory flow and of pressure changes within the WBP chamber induced by changes in gas humidification and temperature from air movement between the chamber and the lungs. The pressure difference between the recording and reference chamber was measured with a differential pressure transducer. The pressure signal was amplified, filtered, recorded, and analyzed with data analysis software (Biosystem XA for Windows, SFT3410 230 ver 2.9; Buxco Electronics, Wilmington, NC) generating tidal volume (V_T_, ml) and breathing frequency (f, breaths min^−1^). Minute ventilation (V_E_, ml min^−1^, BTPS) was determined as a product of tidal volume and breathing frequency. V_T_ and V_E_ were normalized to body weight (ml kg^−1^ and ml kg^−1^ min^−1^, respectively). All experiments were performed at room temperature (24–26 °C). Each rat was placed in the chamber (4.7 L) and left for 30 min of adjustment, while flushing with fraction of atmospheric air at 2.5 L min^−1^ to prevent CO_2_ accumulation. After 30 min, the gas mixture (atmospheric air) in the chamber was switched to hypoxic mixture (8% of oxygen in 92% of nitrogen). Gas equilibration time in the chamber took 50–60 s from a gas switch. After that time hypoxic test consisting of 3 min of breathing with hypoxic mixture was registered.

### High-performance liquid chromatography (HPLC) analysis. Assay of dopamine, serotonin, noradrenaline and metabolites

Fourteen days following 6-hydroxydopamine or vehicle injection to the MFB the animals were euthanized with overdose of pentobarbital sodium and their brains were instantly removed. The left and right striatum and brainstem were dissected. The brainstem was then cut on the left and right sides. Each tissue sample was weighed and frozen (−80 °C) until further biochemical analysis.

Brain tissue was sonicated in ice-cold 0.1 M HClO_4_ solution and centrifuged (13000 rpm, 15 min at 4 °C) to precipitate proteins. The supernatant was filtered (0.2 μm pore size filter; Whatman, USA). The total tissue content of dopamine and 3, 4-dihydroxyphenylacetic acid (DOPAC), serotonin and its metabolite: 5-hydroxyindolacetic acid (5-HIAA), noradrenaline (NA) and its metabolite: 3-methoxy-4-hydroxyphenylglycol acid (MHPG), were analyzed using high performance liquid chromatography with electrochemical detection (HPLC-ED) (L-3500 detector; Merck, Germany) comprising a glassy carbon electrode. The electrochemical potential was set at 0.8 V with respect to an Ag/Ag Cl reference electrode. The aliquots (20 μl) were separated on the column C – 18 (250 × 4.6 mm reverse phase; Nucleosil, 5 μm, Macherey–Nagel, Germany). The mobile phase consisted of 32 mM sodium phosphate (Sigma-Aldrich, USA), 34 nM citric acid (Sigma-Aldrich, USA), and 1 mM octane sulfonic acid (Sigma-Aldrich, USA), and 54 μM ethylenediaminetetraacetic acid (EDTA; Sigma-Aldrich, USA) was added to 18.3 mΩ purified water containing 12% methanol (Merck, Germany). The mobile phase flow rate was maintained at 0.8 ml min^−1^. Samples were quantified by comparing with the standard (Sigma-Aldrich, USA) using ClarityChrom software (Knauer, Germany) using an external standard calibration. The contents of monoamines and parallel metabolites were expressed as pg mg^−1^ fresh tissue.

### Behavioral testing (cylinder test)

Each rat was tested twice, before lesion and two weeks after. A forelimb use asymmetry test was performed to evaluate the effectiveness of lesion evoked with 6-OHDA injection, as previously described [10]. The cylinder test was used to assess the degree of forepaw asymmetry. The rat was placed in a transparent cylinder of 20 cm diameter and 30 cm height. The number of each forepaw contacts to the cylinder wall was recorded and counted for a period of 5 min. The behavioral changes were quantified based on the independent use of either the left or right forelimb to contact the wall during landing and rearing.

### Data and statistical analysis

The each individual value of tidal volume, minute ventilation and respiratory rate were determined by averaging the variables measured for 10s of normoxic and post-hypoxic respiration at chosen time points: 30s, 90s, 120 s, 150 s, 180 s and at 300 s (recovery after hypoxia). To show magnitude of hypoxic response in both neurological states the data were expressed as % of post-hypoxic change with reference to the control normoxic value treated as a 100%. Numbers are reported as mean ± standard error of mean. The experimental data were analyzed using two-way ANOVA (analysis of variance), followed by repeated measurements with defined time points (prior to and after hypoxia) and either injury status (before and after lesion), or pre-treatment with serotonergic agents (prior to and after treatment), as a between condition factor. Differences between individual time points and experimental conditions were evaluated using the Newman-Keuls post-hoc test. To assess differences in the contents of 5-HT, DA, NA and theirs metabolites between groups (SHAM, 6-OHDA), a nonparametric Mann-Whitney U-paired test was used. All values were considered as statistically significant at *P* < 0.05. Statistical analysis was performed using STATISTICA (StatSoft, Poland).

## Results

### Hypoxic ventilatory response in sham and 6-OHDA lesioned rats

**Fig. 2:**
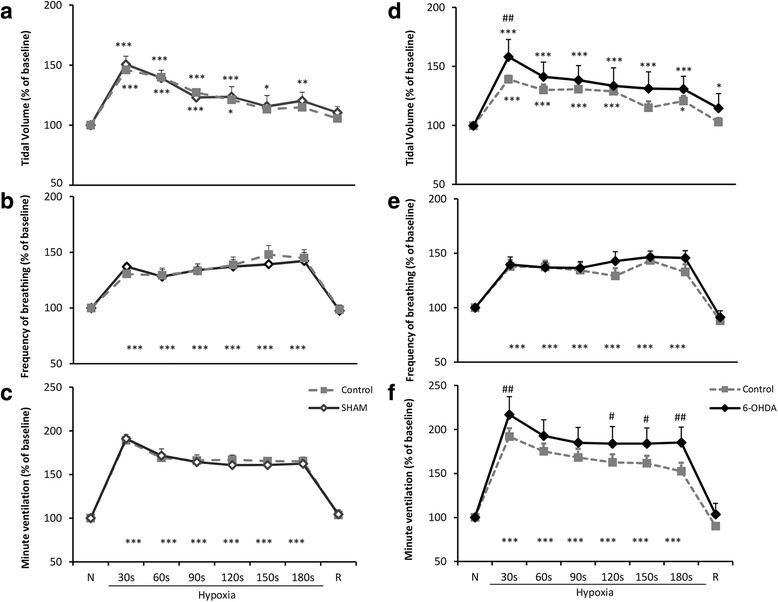
Effects of vehicle (SHAM) or 6-OHDA injection to the MFB on hypoxic respiratory responses of V_T_ (**a**, **d**), respiratory rate (**b**, **e**) and minute ventilation (**c**, **f**). The data are expressed as percentage of change in reference to normoxic value considered as 100%. All values are means ± SEM. *P < 0.05, **P < 0.01,***P < 0.001, *versus* the respective pre-hypoxic value; ^*#*^ *P* < 0.05, ^##^ *P* < 0.01, *versus* the corresponding pre-lesion value; (*n* = 5 (vehicle group), *n* = 9 (6-OHDA group))

Figure 2 shows responses of respiratory variables to hypoxic stimulus before and after sham (a,﻿ b, c) and 6-OHDA (d,﻿ e, f) lesion. The data were expressed as percentage of post-hypoxic change with reference to the normoxic values treated as a 100%. Administration of vehicle to MFB had no influence on normoxic values of V_T_, f and V_E_ (data not shown). Magnitude of ventilatory response to hypoxia displayed no significant differences prior to and after sham lesion (Fig. 2a, b, c).

An unilateral injection of 6-OHDA to MFB reduced normoxic values of frequency of breathing and minute ventilation but did not alter control values of tidal volume (data not shown). However, as we have displayed previously [10] significant influence of nigrostriatal pathway degeneration on the increased respiratory response to hypoxia was noticed (Fig. 2d, e, f). The augmentation of tidal volume response to hypoxia was present at 30 s in comparison to control pre-lesion state (Fig. 2d).

The initial growth of minute ventilation to hypoxic stimulus present at 30s was significantly magnified in 6-OHDA treated state (above 25%). A significant difference in V_E_ between pre-lesion and post-lesion state appeared at 30 s and from 120 s till 180 s (Fig. 2f).

### Involvement of 5HT_2_ receptors in normoxic and hypoxic breathing in rats before and after implementation of the PD model

**Fig. 3:**
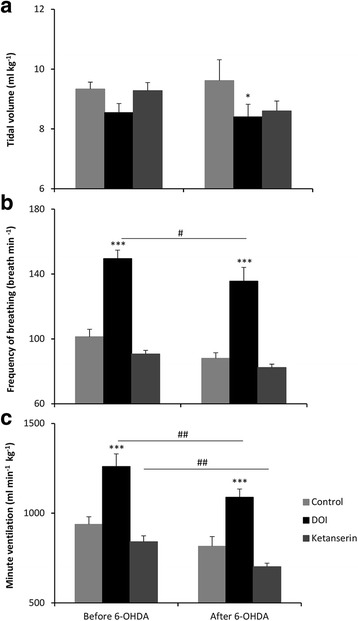
Effects of i.p. injection of DOI and ketanserin on tidal volume (**a**), frequency of breathing (**b**) and minute ventilation (**c**) in normoxic condition in the intact rats and two weeks after lesion with 6-OHDA. All values are means ± SEM. *P < 0.05 *versus* the respective control value; ***P < 0.001, *versus* the respective control and post-ketanserin values. ^*#*^ *P* < 0.05, ^##^ *P* < 0.01, *versus* the corresponding value before and after 6-OHDA treatment (*n* = 9)

Figure 3a, b, c depicts control normoxic respiratory values after DOI and ketanserin injection in pre- and post-lesion states. In normoxia DOI treatment produced diminished tidal volume and increased frequency of breathing and minute ventilation before and after 6-OHDA injection (Fig 3a, b, c). Subsequent ketanserin administration reverted respiratory variables to control values.

**Fig. 4:**
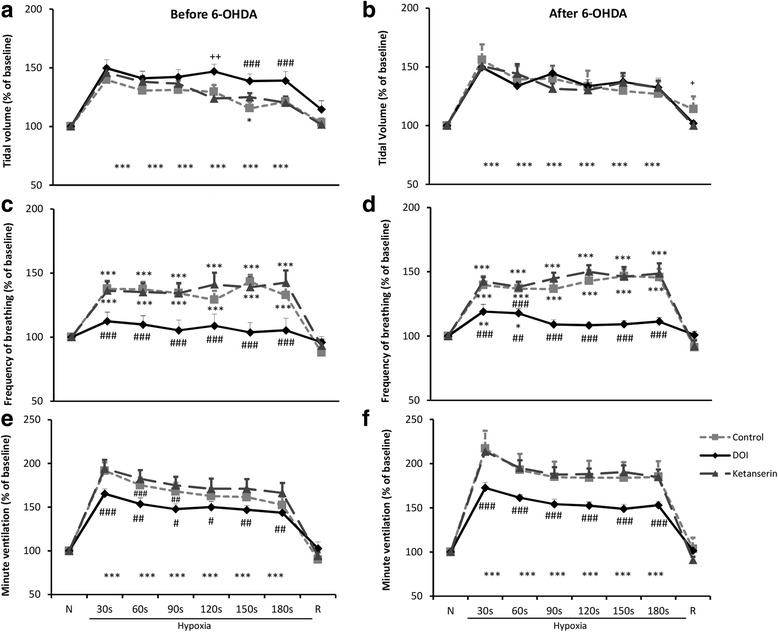
Effects of i.p. injection of DOI and ketanserin on tidal volume response to hypoxia in the intact rats (**a**) and two weeks after lesion with 6-OHDA (**b**). Effects of i.p. injection of DOI and ketanserin on frequency of breathing response to hypoxia in the intact state (**c**) and two weeks after lesion (**d**). Effects of i.p. injection of DOI and ketanserin on minute ventilation response to hypoxia in the intact rats (**e**) and two weeks after lesion with 6-OHDA (**f**). The data are expressed as percentage of change in reference to normoxic value considered as 100%. All values are means ± SEM. *P < 0.05, ***P < 0.001, *versus* the respective pre-hypoxic baseline value; ^#^ *P* < 0.05, ^##^ *P* < 0.01, ^###^ *P* < 0.001, *versus* the corresponding control and post-ketanserin value; ^+^ *P* < 0.05, ^++^ *P* < 0.01, *versus* the corresponding post-ketanserin value (*n* = 9)

DOI application had no effect on magnitude of V_T_ respiratory response to hypoxia before and after lesion (Fig. 4a, b). Frequency of breathing during hypoxia was significantly increased at 30 and 60s after DOI in PD rats only (Fig. 4c, d). Post-DOI response of respiratory rate to hypoxia was diminished by 29% in comparison to control pre-DOI hypoxic response in both neurological states, which was reverted by ketanserin administration (Fig. 4c, d).

Minute ventilation was augmented during hypoxia before and after DOI treatment in pre- and post-lesion states. However, the response to hypoxia was reduced in comparison to pre-DOI state. In the intact state V_E_ in response to hypoxia displayed reduced increase of 17% during the lifespan of stimulus in comparison to control hypoxia. After 6-OHDA lesion to MFB DOI evoked 32% decrease of minute ventilation response to hypoxia (Fig. 4e, f).

### Concentration of dopamine, serotonin, noradrenaline and metabolites in the striatum and brainstem

HPLC analysis confirmed substantial reduction of dopamine and DOPAC concentration in the right lesioned striatum in comparison to left striatum and both striata of sham group. In the brainstem the content of dopamine and its metabolite in ipsilateral and contralateral sides was not disparate (Table 1). There were no dissimilarities in noradrenaline contents in striatum and brainstem between 6-OHDA and vehicle injected animals (Table 1).

**Table 1:** Concentration of dopamine (DA), 3,4-dihydroxyphenylacetic acid (DOPAC), noradrenaline (NA), and 3-methoxy-4-hydroxyphenylglycol (MHPG) in respective left (L) and right (R) brain structures (striatum and brainstem) in sham and 6-OHDA treated rats evaluated by HPLC detection ex vivo

Brain structure		Dopamine				Noradrenaline			
		DA		DOPAC		NA		MHPG	
		Sham	6-OHDA	Sham	6-OHDA	Sham	6-OHDA	Sham	6-OHDA
Striatum	Left	9132 ± 599	7737 ± 1301	3665 ± 210	4956 ± 818	17.04 ± 1.4	18.00 ± 2.0	23.60 ± 3.9	11.71 ± 1.9
	Right	**8314** ± 861	**63.7** ± 72.2*	**3662** ± 264	**5.37** ± 6.2*	26.63 ± 8.6	16.27 ± 7.0	32.52 ± 8.8	13.43 ± 1.4
Brainstem	Left	40.20 ± 6.7	46.42 ± 5.2	29.51 ± 5.8	50.55 ± 3.9	623.81 ± 18.4	575.52 ± 15.4	1.76 ± 0.4	0.99 ± 0.2
	Right	40.40 ± 4.5	29.81 ± 13.2	33.83 ± 3.4	27.89 ± 13.1	617.09 ± 20.1	573.21 ± 21.1	1.19 ± 0.3	1.02 ± 0.2

Data are expressed as mean ± SEM (in pg/mg). Significantly different values are in bold. ^*^ *P* < 0.05, comparison between sham and 6-OHDA groups

**Fig. 5:**
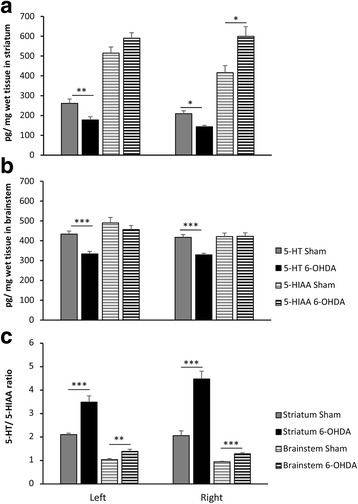
Concentration of serotonin (5-HT), 5-hydroxyindolacetic acid (5-HIAA) (**a**, **b**) and 5-HIAA/5-HT turnover (**c**) in respective *left* (L) and *right* (R) brain structures (striatum and brainstem) in sham and 6-OHDA treated rats evaluated by HPLC detection ex vivo. All values are means ± SEM. *P < 0.05, **P < 0.01, ***P < 0.001, comparison between sham and 6-OHDA treated groups (*n* = 8–9/per group)

Unilateral injections of 6-OHDA into the right MFB produced conspicuous reduction of 5-HT level (32%) in right striatum in compare to tissue of sham operated rats. This was accompanied by 30% increase in the content of serotonin metabolite: 5-HIAA and significant increase of 5-HIAA/5-HT turnover at about 2.2 times in collation to vehicle injected striatum (Fig. 5a, c).

Average concentration of 5-HT in the left intact striatum of 6-OHDA treated group was also diminished by 32% in compare to left striatum of sham rats. Turnover of 5-HIAA/5-HT was increased by 1.6 times, however the level of 5-HIAA was not significantly distinct from the left striatum of sham lesioned rats (Fig. 5a, c).

In the brainstem of 6-OHDA lesioned animals bilateral reduction over 20% of serotonin was observed, compared to sham group. This resulted in 1.3 higher 5-HIAA/5-HT turnover in both sides of the brainstem of 6-OHDA treated animals (Fig. 5b, c).

### Cylinder test

**Fig. 6:**
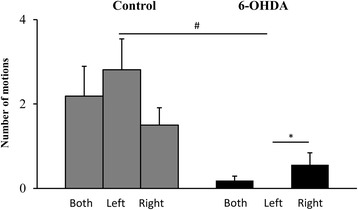
Effects of 6-OHDA injection on incidence of ipsilateral and contralateral to lesion forepaw usage. 6-OHDA application caused preference to use the right forelimb, ipsilateral to lesioned right MFB during rearing and landing in the cylinder test. All values are means ± SEM. *P < 0.05, *versus* the use of *left* or *right* forepaw in 6-OHDA treated state; ^*#*^ *P* < 0.05, between pre- and post-lesion states

Sham lesioned animals displayed no signs of asymmetry and similar level of activity in comparison to pre-injection state (data not shown). 6-OHDA lesioned rats showed locomotor asymmetry, expressed by preference usage of the right forepaw, ipsilateral to the site of neurotoxin injection (Fig. 6). Before lesion in the intact state the animals touched the cylinder wall with right or left forepaw proportionate number of times.

## Discussion

We showed for the first time that alteration of serotonin system provoked by unilateral injection of 6-OHDA into the rat MFB affects hypoxic ventilatory response after stimulation of 5-HT_2_ receptors. The most interesting finding was correlation of serotonin level decline in striatum and brainstem with decreased response of minute ventilation to hypoxic stimulus after DOI injection.

6-hydroxydopamine injection to the MFB produced over 90% depletion of dopamine in the striatum ipsilateral to the site of the lesion, which we have shown also previously [10]. This time we displayed no alteration in noradrenaline concentration and over 30% depletion of serotonin in both striata and over 20% in both sides of the brainstem.

Similar to our results, reduced level of serotonin and increased turnover of 5-HIAA/5-HT in the brainstem was described two weeks after intracerebroventricular injection of 6-OHDA [21]. Higher change in striatal 5-HT content (50%) and ratio of 5-HIAA/5-HT was found in the MFB model, but after 6 weeks of neurotoxin application [22]. Metabolism of serotonin is attained almost exclusively via the monoamine oxidase and aldehyde dehydrogenase action, resulting in the formation of terminal metabolite 5-HIAA. Decline in 5-HT level in striatum and brainstem and augmented ratio of 5-HIAA/5-HT particularly in striatum, points to increased metabolism of serotonin. This metabolic enhancement is officially considered as a compensatory mechanism or neurochemical adaptation, the result of which is to restore monoaminergic balance during the initial phase of synaptic degeneration. Intensity and duration of such adaptive reaction are strictly related to metabolic reserve of injured neurons, even beyond the dopaminergic tract [23].

This was revealed earlier that in Parkinson’s disease and experimental models of PD apart from dopaminergic pathways, also serotonergic system is liable to alterations [24–26]. Degeneration of dopaminergic neurons caused by 6-OHDA injection may have impact on brain level of serotonin, electrophysiological characteristics of serotonergic neurons and functioning of serotonergic receptors [12, 27].

Serotonin is important modulator of respiratory rhythm and pattern, which was documented by research on transgenic mice lacking serotonergic neurons. The animals displayed depressed ventilation and high frequency of severe apnea [28]. Deficiency of serotonin in the nucleus of solitary tract is reckoned to be related to pathogenesis of sudden infant death syndrome (SIDS) [29]. The role of serotonin in ventilatory response to hypoxia remains undefined. Some studies pointed that caudal medullary raphe and its serotoninergic neurons are not involved in mediating respiratory response to hypoxia [30] and that absence of serotonin in brains of genetically modified mice has no impact on magnitude of ventilatory long term facilitation following intermittent hypoxia [31]. On the other hand, serotonin released in the area of the dorsomedial medulla oblongata containing NTS and nucleus of hypoglossal nerve participated in initiation of hypoxic ventilatory response and its depressive phase [18]. Antagonism of 5-HT_2_ receptors in this localization produced delayed post-hypoxic hyperventilation and reduced depression phase [18].

Neuronal degeneration and dystrophic neurites in serotonin-producing raphe nuclei has been evidenced in Parkinson’s disease [32, 33]. Medullary raphe region provides 5-HT innervation to the NTS [14], which receives afferents from chemoreceptors of the carotid body and sends direct efferent connections to respiratory motor neurons influencing ventilatory response to hypoxia [34]. Serotonergic boutons contacting cat NTS neurons and phrenic motoneurons were immunohistochemically demonstrated previously [35, 36].

We hypothesize that changes in serotonergic system playing role in regulation of breathing contribute to respiratory dysfunction present in PD such as dyspnea, sleep apnea or altered ventilatory response to hypoxia [4, 5, 7, 8]. Thus, the present study attempted to investigate the impact of nigrostriatal pathway degeneration on breathing in normoxia and during hypoxic stimulus after intraperitoneal administration of blood-brain barrier permeating agonist and antagonist of 5-HT_2_ receptors; DOI and ketanserin, respectively. DOI injection in both neurological states: before and after lesion stimulated respiratory rate and minute ventilation in normoxia. In hypoxia decrease of response was observed for both parameters in comparison to control pre-DOI hypoxic response. Subsequent ketanserin administration inverted normoxic respiration and respiratory response to hypoxia to its control levels. Our results correspond with study by Guner et al. [37], who showed that serotonin applied intracerebroventricularly into chemodenervated rabbits augmented V_T_ and V_E_ in normoxia, however diminished ventilatory response to hypoxia. The authors speculated that the latter could have been initiated by accumulation of dopamine with its inhibitory influence on respiration. It has been shown previously that DOI application augmented release of dopamine, which was reverted by blockade of 5-HT_2_ receptors [38, 39]. So conceivably, in our study the agonist could modulate dopaminergic system via stimulation of dopamine release and indirectly decrease hypoxic ventilatory response. Infusion to striatum compounds inhibiting reuptake of serotonin combined with systemic injection of haloperidol increased release of dopamine [40]. Studied in our research model of PD is parallel to the state present after haloperidol, which is considered as one of models of Parkinson’s disease [41]. Thus, reduced response of HVR in unilaterally lesioned rats could be the result of increased dopamine release in the intact hemisphere.

Another plausible explanation of substantially diminished response of ventilation in response to hypoxia after DOI (30%) in compare to HVR in pre-lesion state (17%) could be the loss of balance between dopaminergic and serotonergic system. When 30% depletion of serotonin is accompanied by substantial of dopamine, activity of serotonergic system may predominate. Therefore reduced HVR after DOI in lesioned rats corresponds with inhibitory action of endogenous and exogenous 5-HT on depressive phase of ventilatory response to hypoxia [18, 30] and inverse stimulatory activity of 5-HT_2_ receptors’ antagonist on hypoxic activity of phrenic nerve [42].

There are reports showing elevated level of 5-HT_2A_ receptor mRNA in the striatum and subthalamic nucleus in the same unilateral MFB model of PD [43, 44]. It is possible that similar upregulation of serotonin receptors could take place also in our study. To our knowledge no studies dealing with expression of brainstem 5-HT_2_ receptors in PD have been performed. Nevertheless, we cannot exclude that DOI is more potent in inhibiting hypoxic response in our lesioned animals because of upregulation of 5-HT_2_ receptors.

Tuppy et al. [45] evidenced that bilateral intrastriatal 6-OHDA injection evoked substantial reduction in neurons in the ventral respiratory group (retrotrapezoid nucleus, pre-Botzinger complex and rostral ventral respiratory group), which correlated to the diminished tachypneic response of frequency of breathing to hypercapnia. Although they studied PD model after three times longer period of time we cannot rule out that loss of respiratory neurons could appear in our study and participate to reduced HVR after activation of 5-HT_2_ receptors.

Further studies are needed to define the role of serotonergic system in altered hypoxic breathing in Parkinson’s disease. Probably the use of bilateral model and examination of the expression of 5-HT_2_ receptors in the brainstem would be more conclusive. An addressing the role of another serotonin receptor in PD, like 5-HT_3_, which seems to contribute to pathogenesis of SIDS, could be also very important issue [30].

## Conclusion

Here, we demonstrated that 6-OHDA induced damage of the nigrostriatal pathway affects serotonergic system, which was manifested by reduced levels of serotonin in the striatum and brainstem and decreased respiratory response to hypoxia after activation of 5-HT_2_ receptors. It implies that depletion of serotonin in the brainstem may contribute to respiratory anomalies present in PD.

## Abbreviations

5-HT: Serotonin
6-OHDA: 6-hydroxydopamine
ANOVA: Analysis of variance
DA: Dopamine
DOI: 2,5-dimethoxy-4-iodoamphetamine
HPLC: High-performance liquid chromatography
HVR: Hypoxic ventilatory response
MFB: Medial forebrain bundle
NTS: Solitary tract nucleus
PD: Parkinson’s disease
SIDS: Sudden infant death syndrome
SN: Substantia nigra pars compacta
